# Influence of fruit dispersal on genotypic diversity and migration rates of a clonal cactus from the Chihuahuan Desert

**DOI:** 10.1002/ece3.4657

**Published:** 2018-12-07

**Authors:** Erick García‐Morales, Israel G. Carrillo‐Ángeles, Jordan Golubov, Daniel Piñero, María C. Mandujano

**Affiliations:** ^1^ Laboratorio de Genética y Ecología Instituto de Ecología Universidad Nacional Autónoma de Mexico Mexico City México; ^2^ Escuela de Biología Universidad Autónoma de Querétaro Mexico City Mexico; ^3^ Laboratorio de Ecología, Sistemática y Fisiología Vegetal Departamento El Hombre y Su Ambiente‐CBS‐Universidad Autónoma Metropolitana‐Xochimilco Mexico City Mexico

**Keywords:** establishment, genotype, migration, population structure, ramet

## Abstract

The diverse offspring of clonal species differ in their dispersability, influencing genotypic diversity and clonal structure. Here, we determined dispersal patterns and their impact on genetic structure in *Opuntia microdasys*, a self‐incompatible cactus with three dispersal units (one sexual and two clonal). We analyzed dispersal, using experiments at three populations, and assessed multilocus genotypes (ISSR markers) of all individuals in 10 clumps per population with known reproductive origin (sexual or clonal). Genotype of all samples, population structure, and migration between clumps and populations were assessed with GenAlEx and GenoDive, assuming higher genotypic diversity and migration when sexual reproduction is more frequent. We determined the most likely number of genetic clusters with STRUCTURE and geneland. Dispersal differed among populations; primary dispersal occurred at short distances and was farthest on steep slopes, and dispersal distance increased after secondary dispersal. Clumps had 116 different multilocus genotypes in three spatially explicit genetic clusters. We detected genetic structure at small scale, genotypic diversity among clumps varied between populations; diversity decreased while clonal dominance increased, and the most variation occurred among clumps. Genetic structure was moderate, suggesting gene flow by seed dispersal allows slight differentiation among population at large scales. Genetic diversity within clumps was the lowest because dispersal of clonal propagules was limited and caused genotypic dominance at local scale. However, the combined dispersal pattern of sexual and clonal dispersal units is fine‐tuned by environmental factors, generating a range of genetic diversity among clusters and populations. This pattern suggests that genetic structure of clonal plants is more dynamic than thought, and dispersal of different types of offspring affects genetic structure at many scales.

## 1 INTRODUCTION

Dispersal is a crucial process for the maintenance (in space and time) of spatially structured populations (Nathan & Muller‐Landau, 2000; Ronce, 2007), as it affects both local populations and the entire distribution of species. The movement of reproductive structures to suitable sites and the patterns of recruitment affect the spatial arrangement of individuals (Bullock, Shea, & Skarpaas, 2006; Dean & Milton, 2000; Nanami, Kawaguchi, & Yamakura, 1999; Nathan & Muller‐Landau, 2000; Pairon, Jonard, & Jacquemart, 2006), the genetic diversity of populations (Oddou‐Muratorio, Klein, Vendramin, & Fady, 2011; Pairon et al., 2006; Ronce, 2007), and the geographic distribution of species (Chambers & MacMahon, 1994; Marco, Montemurro, & Cannas, 2011). Spatial patterns of the individuals may also drive future biotic interactions (Chambers & MacMahon, 1994).

Most studies on plant dispersal have focused on pollen or seeds, that is, sexual diaspores (Geng et al., 2008) and mostly ignored other types of dispersal units (Ronce, 2007), even though most perennial plants combine sexual reproduction with some form of clonality (i.e., mixed reproduction, Arizaga & Ezcurra, 2002; Barrett, 2015; Bullock, Shea, & Skarpaas, 2006; Mandujano, 2007; Oddou‐Muratorio, et al., 2011). There are multiple strategies for clonality (Arizaga & Ezcurra, 2002; Bullock et al., 2006; Klimeš, Klimešová, Hendriks, & van Groenendael, 1997; Mandujano, 2007), and in some cases, a sexual structure could also act as a clonal diaspore (Klimešová & Klimeš, 2008). Several species of Cactaceae can display different modes of clonality, for example, the stems of *Ferocactus robustus* (Carrillo‐Angeles, Mandujano, & Golubov, 2011), all species of *Cylindropuntia* (chollas) from the Sonoran Desert (Bobich & Nobel, 2001), and *Echinopsis thelegona* (Ortega‐Baes & Gorostiague, 2013) break the connection with the parent plant acquiring independence. Failures in fruit development cause fruit abortion (Bravo‐Hollis, 1978; Fuentes Pérez, 2008; Negron‐Ortiz & Strittmatter, 2004; Nobel, 2002; Piña, Montaña, & del Mandujano, 2007; Vázquez‐Delfín, Sánchez‐Serrano, & Martorell – Delgado, 2005), which also may trigger clonality through pseudo‐viviparity (i.e., clonal offspring—plantlets—are produced by failed sexual structures) (Charpentier, 2002; Ellstrand & Roose, 1987; Elmqvist & Cox, 1996; Gélin et al., 2017; Plasencia‐López, 2008), for example, plantlets are commonly developed in *Cylindropuntia leptocaulis* (Vázquez‐Delfín et al., 2005) and *Opuntia microdasys* (Palleiro, Mandujano, & Golubov, 2006).

Clonal propagules and sexual diaspores differ in morphological and physiological traits, and in dispersal capabilities (Mandujano, 2007; Zhang & Zhang, 2007). For example, in *Prunus serotina,* either the presence or lack of mesocarp determines the dispersal vector and, in consequence, the dispersal curves; when the mesocarp is present, seed dispersal by gravity occurs up to 5 m around the source, but when the mesocarp is absent, seeds are dispersed by birds up to 30 m away from the source (Pairon et al., 2006). Because clonal propagules often lack specialized dispersal structures, dispersal is assumed to be limited (Bullock et al., 2006; Eckert, 2002; Winkler & Fischer, 2002). In species with mixed reproduction (i.e., combined sexual and clonal recruitment), the spatial genetic structure has two opposite patterns (Alberto et al., 2005), either dispersal and subsequent establishment promote the spatial arrangement of intermingled ramets of different genets (i.e., multiclonal patches) or limited dispersal of clonal propagules (Bobich & Nobel, 2001; Bravo‐Hollis, 1978; Fuentes Pérez, 2008; Negron‐Ortiz & Strittmatter, 2004; Nobel, 2002; Piña et al., 2007) leads to groups of clumped ramets of the same genet (i.e., genotypic dominance in monoclonal stands [superclones]) (Alberto et al., 2005; Barrett, 2015; Charpentier, 2002; Gélin et al., 2017). On one hand, dispersal by direct observation is plausible for species with large and easily traceable dispersal units; for these cases, mark and track experiments are useful to determine the source of clonal propagules. Although direct methods provide exact information on dispersal distances, the difficult task of gathering data for dispersal over long distances poses a serious limitation (Bullock et al., 2006; Nathan & Muller‐Landau, 2000; Nathan, Perry, Cronin, Strand, & Cain, 2003). In addition, in species with high clonal recruitment, it is hard to determine the source of a ramet, as some species tend to be dominated by a superclone (Bravo‐Hollis, 1978). On the other hand, indirect methods that use molecular markers are well developed to evaluate effective dispersals (i.e., dispersal plus establishment events; Cain, Milligan, & Strand, 2000, Levin, Muller‐Landau, Nathan, & Chave, 2003) and determine the number and distance of migrants per generation and the degree of genetic structure and differentiation between populations (Cain, Milligan, & Strand, 2000; Carrillo‐Angeles et al., 2011; Levin, et al., 2003; Manel, Gaggiotti, & Waples, 2005; Pritchard, Stephens, & Donnelly, 2000a). Indirect methods, however, are focused on effective dispersal (Cain et al., 2000) and exclude all the reproductive structures that dispersed but have not established or survived.

Our study species, *Opuntia microdasys* (Cactaceae), is a clonal cactus that produces three kinds of offspring, one of sexual origin (seedlings from seeds formed from ripe fruits: sexual diaspores) and two of clonal origin (detached cladodes that take root and unripe fruits that can form new plantlet recruits; Palleiro et al., 2006). Frequent short‐distance dispersal of clonal diaspores will result in spatial aggregation of clone mates (Bobich & Nobel, 2001; Bravo‐Hollis, 1978; Fuentes Pérez, 2008; Negron‐Ortiz & Strittmatter, 2004; Nobel, 2002; Piña et al., 2007). Morphological and demographic differences among clonal and sexual diaspores of *Opuntia microdasys* provide an interesting model to assess the dispersal of sexual and clonal diaspores. Palleiro et al. (2006) found that the plantlets mainly establish under the canopy of adults individuals no more than ca. 90 cm from the parent, forming clusters of new offspring under the canopy of adults plants (i.e., clumps of plants). But not only clusters of clonal propagules become established; Dean and Milton (2000) found clusters of intermingled genets of *Opuntia ficus‐indica* around telegraph poles and wire fences from seeds dispersed by crows. The demographic contributions of each type of propagule (Palleiro et al., 2006) and the spatial configuration of genotypes (Carrillo‐Angeles et al., 2011) produced a gradient of clonality and sexuality between populations. Thus, we expected that dispersal promotes the intermingling of ramets of different genets (i.e., multiclonal patches) and higher genetic diversity in the more sexual population and monoclonal clumps with low genetic variation in less sexual populations where clonal diaspores remain in close proximity. Evaluating fruit dispersal should help elucidate whether seeds (ripe fruits) or seedless (unripe) fruits move longer distances from parent plants. In addition, in species with high clonal recruitment, it is hard to determine the source of a ramet, as some species tend to be dominated by a superclone (i.e., over‐representation of ramets with the same multilocus genotype) (Bravo‐Hollis, 1978). Because the interaction between the environment, dispersal availability, and type of dispersal unit imposes a challenge when studying dispersal of a clonal species, we combined direct and indirect methods to assess dispersal patterns of sexual and clonal dispersal units (Bullock et al., 2006; Nathan et al., 2003).

Here, we aimed (a) to determine the spatial genetic structure that results from dispersal and establishment events of either sexual diaspores or clonal propagules and (b) to determine the genotypic diversity and migration rate within and between both clumps of plants and populations of *O. microdasys* (Cactaceae) in the southern Chihuahuan Desert.

## 2 MATERIALS AND METHODS

### 2.1 Study species

**Figure 1:**
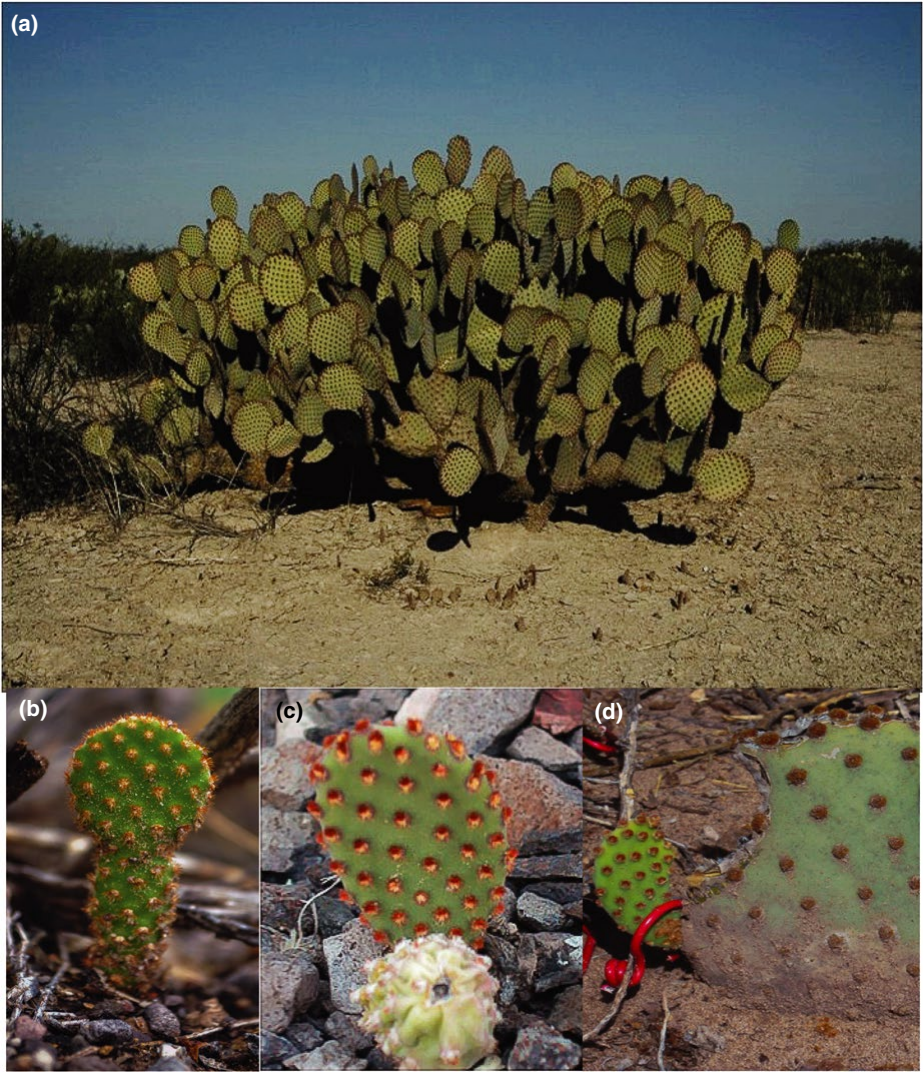
*Opuntia microdasys* (a) and the three different types of recruits originating from different propagules: (b) seedling from seeds, (c) ramets from cladodes, and (d) plantlets from aborted fruits

*Opuntia microdasys* (Lehm.) Pfeiff. (Cactaceae; Figure 1a), bunny ears or blinding prickly pear, is a self‐incompatible, clonal cactus that forms shrubs up to 1 m tall, with oval, bright green cladodes (racket‐like stems) that lack spines (Bravo‐Hollis, 1978). Areoles have numerous reddish brown or yellow glochids. The segments of the perianth in the flowers are yellow, with flowering between April and May. The fruits are globose, fleshy, 2–2.5 cm in diameter, and turn from green into red when mature, usually ripen between June and August. Unripe and mature fruits either disperse by gravity or are removed by birds and mammals (E. García‐Morales, personal field observations). When the fruits reach the ground, several factors could influence their dispersal, but the immediate factors are the slope and microtopography of the site. Other agents such as temporary streams can move fruits on the ground farther during a heavy rain (M. Mandujano, unpublished data).

*Opuntia microdasys* usually grows on sandy to loamy calcareous soils in hills and uplands in the Chihuahuan Desert of Mexico. The species reproduces both clonally and sexually (Palleiro et al., 2006; Piña et al., 2007). The flowers are pollinated by solitary bees (*Diadasia* sp.), and the species is self‐incompatible (Piña et al., 2007) because all new genets are recruited from seeds produced from cross‐fertilization (Figure 1b). Failure of fruit development causes fruit abortion (Piña et al., 2007); aborted unripe fruits are common propagules (94.3% of the recruits in the Mapimi Biosphere Reserve [MBR] are plantlets; Zhang & Zhang, 2007; Cain et al., 2000) that could recruit new clonal individuals called plantlets (Figure 1c). Two characteristics of the sexual structures in the Cactaceae family are distinctive from those of all other angiosperms and potentially allow clonality via aborted fruits: The axillary buds in areoles differentiate into new stems, flowers, spines, glochids, etc. (Bravo‐Hollis, 1978; Nobel, 2002), and the ovary is covered by a modified stem (Nobel, 2002) called the pericarpel (Bravo‐Hollis, 1978). Because of its vegetative nature, the pericarpel is green and covered by areoles early in the development of the flower and fruit (Bravo‐Hollis, 1978; Fuentes Pérez, 2008). Areoles of the pericarpel most commonly produce spines, scales, and wool (Bravo‐Hollis, 1978). In some cases, however, they produce leaves (*Pereskia*), new stems (*Cylindropuntia leptocaulis*; Winkler & Fischer, 2002), or new flowers (*Opuntia prolifera, Cylindropuntia bigelovii, Cylindropuntia fulgida*; Montaña, 1990, and *Consolea corallicola*; Alberto et al., 2005). These examples highlight the fact that, sometimes, sexual structures in Cactaceae also act as clonal diaspores, (i.e., plantlets, Figure 1d). In addition, *O. microdasys* can form physiologically independent ramets from detached cladodes (Figure 1d; Palleiro et al., 2006). Consequently, clonal recruitment appears to be important for populations of *O. microdasys* in the MBR, and clonal propagules are spatially autocorrelated with adult clones within a radius of 20 m (Carrillo Angeles, Golubov, Milligan, & Mandujano, 2011).

### 2.2 Study site

**Figure 2:**
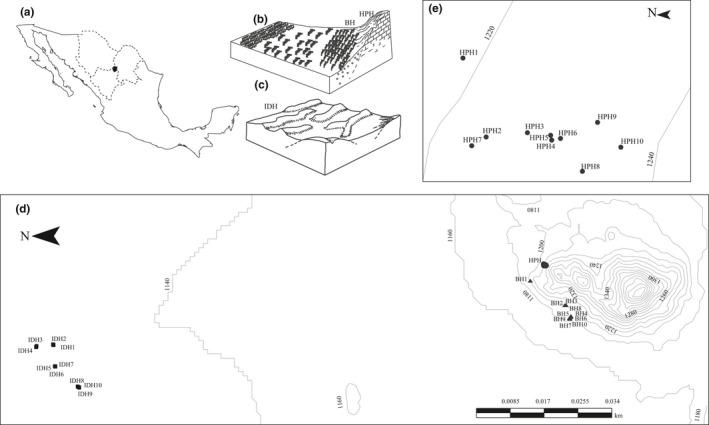
Representation of the landforms inhabited by *Opuntia microdasys* in Mapimi Biosphere Reserve. Contiguous habitats in the San Ignacio Mountains: (a) Hill‐Piedmont (circles), Bajada (triangles), and Interdune (squares), (b) close‐up of Hill‐Piedmont habitat (note scale differences)

The study was conducted in three populations of *O. microdasys* at MBR—Bajada (BH), Hill‐Piedmont (HPH), and Interdune (IDH; Figure 2)—in the southern Chihuahuan Desert, Mexico (26**°**29′–26**°**52′N and 103**°**32′–103**°**58′W, 1,100 m above sea level, 20.8**°**C mean annual temperature, and 264 mm mean annual rainfall, of which 80.2% falls between June and October, Figure 2a). The HPH and BH are contiguous populations located on the west side of San Ignacio Mountain (Figure 2b) with steeper slopes of >10% and 2%, respectively. In the HPH, shallow, stony soils overlay igneous rock, and the sparse vegetation is dominated by *Fouquieria splendens* Engelm., *Larrea tridentata* (Sessé & Moc. ex DC.) Coville, and *Yucca rigida* (Engelm.) Trel. The soils are deep in BH, usually a mixture of gravel and sand, and the vegetation is dominated by *Larrea tridentata*,* F. splendens,* and *Opuntia rastrera* Weber. The IDH has sandy and deep soils with slopes of <1%; the dunes form a network of hills connected by flat interdune plains where the dominant vegetation includes *Acacia constricta* Benth., *Acacia greggii* A. Gray, *L. tridentata,* and *Prosopis glandulosa* Torr.

Palleiro et al. (2006) reported different recruitment rates for each type of propagule between populations of *O. microdasys* and represented recruitment as a percentage of offspring that were produced either sexually or clonally. IDH has the highest percentage of establishment by cladodes, BH has the highest percentage of establishment of plantlets, and HPH has the highest percentage of sexual recruitment.

### 2.3 Fruit dispersal

Two field experiments were set up to explore fruit dispersal of *O. microdasys* during fruiting. In the first experiment, we measured primary dispersal, the distance traveled by fruits immediately after they had detached from the parent plant. In the second experiment, we measured the combined effect of primary and secondary dispersal during the entire reproductive season by marking and tracking the fruits.

#### 2.3.1 Primary fruit dispersal

We experimentally simulated fruit dropping from parent plants at each population (BH, HPH, and IDH) to assess the process of primary dispersal of fruits of *O. microdasys* in relation to landscape and their effect in the spatial distribution of plantlets around parent plants. Ten reproductive focal plants were randomly selected within each population, and the area around the plant crown was split into eight quadrants that corresponded to cardinal and intercardinal directions (i.e., N, NE, E, SE, S, SW, W, and NW). An unripe fruit (ca. 2 cm long and 1.7 cm diameter) was taken from a focal plant and painted; the detachment of fruits from the parent plant was simulated by dropping a different painted fruit 40 times in each cardinal direction, from a height of 1 m to mimic the natural fruit detachment. Fruit was released at the tip of the branches to avoid impact with cladodes that could alter their fall strength or trajectory. Once the fruit reached the ground and stopped moving, we measured the distance traveled by the fruit (cm) and recorded the quadrant in which the fruit was located (fate). We analyzed the fruit fate with circular statistics (Fisher, 1996). Correlation between the number of fruits that fell in each quadrant (circular data) and distance reached by fruits (linear data) was analyzed using Oriana v4.02 (Kovach, 2011). Finally, the dispersal distance of fruits was analyzed with a two‐way nested ANOVA (Kutner, Nachtsheim, Neter, & Li, 1996) in JMP 8.0.2 (population and quadrants as fixed factors and focal individual nested within population as random effect) and a Tukey test (Kutner et al., 1996).

#### 2.3.2 Marking and tracking fruits

The fate of fruits was followed over the entire reproductive season (from June to September of 2010) to assess the distances reached by fruits over longer periods. In this experiment, the distance from the parent plant to the point where the fruits were found could have resulted from primary and/or secondary dispersal. Here, we assumed that any fruits found beyond the mean distance from the focal plant measured in the primary dispersal experiment had undergone secondary dispersal.

During the 2010 fruiting season, 10 reproductive focal plants in each population (30 plants in total), with at least 200 fruits each, were selected. Using unique plant‐specific colors, we painted all fruits within each focal plant to later identify the parentage. Once a month, we counted all fruits that remained on the parent plant and all painted fruits found on the ground. We estimated the proportion of detached fruits at each count as the ratio of the number of fruits found on the ground to the number of fruits originally painted on the parent. We recorded the distance from the parent plant, the stage of maturity, and the fate for all painted fruits found on the ground. The fruits were assigned to one of four categories of maturity by direct observations of their morphological qualities: (a) ripe, (b) unripe, (c) aborted, and (d) other (e.g., part of a fruit or painted pericarpel that once covered a fruit). In addition, we identified five possible fates for fruits: (a) parent, when fruits were found under the crown of the focal plant; (b) other plants, when the fruit was observed under the plant crown of other plants in the neighborhood; (c) exposed, when a fruit was found in bare areas; (d) *Neotoma* nest, when the fruit was located in the nest of the white‐throated wood rat (*Neotoma albigula*); and (e) lost, the remaining fruits that were not found after detachment.

With the proportion of detached fruits every month as a response variable and populations (IDH, BH, and HPH) and sampling period (months) as fixed factors, the results were analyzed with a generalized linear model using a binomial distribution and a logit link function in JMP 8.0.2. The distances reached by fruits were evaluated with a two‐way nested ANOVA in JMP 8.0.2, considering population and month as fixed factors and individual nested in population as random effect. The number of fruits at each stage of maturity was evaluated with a generalized linear model with a Poisson distribution of residuals and the log link function in JMP 8.0.2; fruit count was the response variable, with stage of maturity of fruits, populations, and month as factors (Kutner et al., 1996). Finally, the number of fruits for each fate was analyzed using a generalized linear model with a Poisson distribution of residuals and the log link function in JMP 8.0.2; fruit count was the response variable with fates of fruits, populations, and sampling period as fixed factors (Kutner et al., 1996).

### 2.4 Genetic assessment of dispersal

#### 2.4.1 Sample collection

We collected samples of fresh tissue from 10 clumps of plants in each population. A clump of plants consisted of a parent plant (focal) and all their putative offspring established under its crown. Offspring were considered to be any established individual with a size of less than three cladodes; when possible, we recorded whether the offspring originated via unripe fruit, cladode, or seed. All clumps of plants sampled at each population were selected within permanent plots that were previously established for a demographic study that began in 2007. We sampled 577 individual ramets distributed among 30 clumps of plants across the three populations (BH = 347, HPH = 148, IDH = 82; Table 1). Approximately 10 g of fresh tissue from newly produced cladodes was collected from all ramets (physiologically independent individuals). This tissue proved to be most suitable for extraction and amplification of DNA. Each sample was placed in a sterile 5‐cm polyethylene bag with 5 g of silica gel. The silica gel was changed periodically until the tissue was completely dry (this process is required because the *Opuntia* tissue contains mucilage that interferes with DNA extraction). DNA was extracted with a Fast‐DNA Kit (116540600 MP Biomedicals), and we test the quantity and purity with electrophoresis in a 2% agarose gel to obtain between 10 and 20 ng.

**Table 1:** Attributes of plant clumps. Habitat‐Clump ID: population of origin and identity of each sampled clump (BH: Bajada, HPH: Hill‐piedmont, IDH: Interdune, clumps 1–10). Number of individual ramets sampled in the clump including the parent plant (*N*), and number of each type of recruit identified along the sampled (P: plantlet, C: cladode, S: seedling, ?: origin could not be assigned) based on morphological observations. Percentage of polymorphic bands (%P), number of private bands (PB), unbiased genotypic diversity (*R*), unbiased Nei's genetic diversity index (D), and corrected Shannon index for clumps and habitats

Habitat‐Clump ID	*N*	P	C	S	?	%P	PB	*R*	*D*	Shannon
BH1	22	21	0	0	0	0.00	1	0.000	0	0
BH2	37	35	1	0	0	2.87	0	0.028	0.054	0.054
BH3	33	27	0	0	5	7.53	1	0.156	0.333	0.329
BH4	23	16	0	0	6	0.36	0	0.045	0.087	0.078
BH5	22	21	0	0	0	10.04	1	0.048	0.091	0.08
BH6	33	32	0	0	0	16.5	2	0.250	0.432	0.459
BH7	48	46	0	0	1	0.00	0	0.000	0	0
BH8	39	38	0	0	0	0.00	0	0.000	0	0
BH9	47	46	0	0	0	0.00	1	0.000	0	0
BH10	43	42	0	0	0	9.68	1	0.119	0.221	0.237
Total	347	324	1	0	12	57.71	7	0.087	0.907	1.104
HPH1	8	3	0	0	4	11.11	3	0.286	0.464	0.319
HPH2	9	8	0	0	0	2.51	3	0.125	0.389	0.23
HPH3	13	11	0	0	1	15.41	2	0.917	0.987	1.068
HPH4	29	27	0	1	0	10.04	1	0.250	0.48	0.484
HPH5	18	17	0	0	0	0.00	0	0.000	0	0
HPH6	33	32	0	0	0	24.73	5	0.719	0.949	1.266
HPH7	21	15	1	0	4	15.77	3	0.250	0.495	0.453
HPH8	8	7	0	0	0	1.79	3	0.143	0.25	0.164
HPH9	5	4	0	0	0	6.81	2	0.750	0.9	0.579
HPH10	4	1	0	0	2	15.05	1	0.667	0.833	0.452
Total	148	125	1	1	11	82.44	23	0.435	0.949	1.519
IDH1	24	20	1	2	0	13.62	2	0.130	0.239	0.223
IDH2	11	2	0	8	0	9.32	5	0.100	0.182	0.132
IDH3	9	2	1	5	0	16.13	2	0.125	0.222	0.151
IDH4	8	4	0	3	0	0.00	1	0.000	0	0
IDH5	13	3	0	9	0	5.38	1	0.167	0.295	0.233
IDH6	4	0	0	3	0	0.00	4	0.000	0	0
IDH7	4	1	0	2	0	0.00	2	0.000	0	0
IDH8	3	1	1	0	0	5.38	2	1.000	1	0.477
IDH9	3	0	0	0	2	0.00	0	0.000	0	0
IDH10	3	0	2	0	0	0.00	3	0.000	0	0
Total	82	33	5	32	2	65.95	22	0.222	0.884	1.044
All	577	482	7	33	25			0.198		

#### 2.4.2 Molecular analysis

We used intersimple sequence repeats (ISSRs) as molecular markers to genotype each sample (Zietkiewicz, Rafalski, & Labuda, 1994). The use of dominant markers (such as ISSRs or AFLPs) is a common technique used in ecological and systematic studies of plants and other organisms because of their low cost and high reproducibility, variable loci, and distribution throughout the genome (Bornet & Branchard, 2001; Nybom, 2004; Zietkiewicz et al., 1994). Compared with other dominant markers such as RAPDs, ISSRs are advantageous for two reasons: High annealing temperatures in ISSR protocols make PCR conditions more stringent for the amplification of fragments (Nybom, 2004), and the longer primers seem to provide the same reproducibility as microsatellites (Bornet & Branchard, 2001; Nybom, 2004). Preliminary tests were done to standardize the protocols for several primers, and three primers (817 [CAC ACA CAC ACA CAC AA], 827 [ACA CAC ACA CAC ACA CG], and 842 [GAG AGA GAG AGA GAG AYG; Y = C or T]; IUBC SSR first 100‐9, University of British Columbia) yielded consistent banding patterns and polymorphism for *O. rastrera* (Plasencia‐López, 2008) and *O. microdasys* (Carrillo‐Angeles et al., 2011). The three selected ISSR primers amplified 281 loci with reproducible bands, which were used to assess the multilocus genotype of all sampled *O. microdasys* individuals.

**Figure 3:**
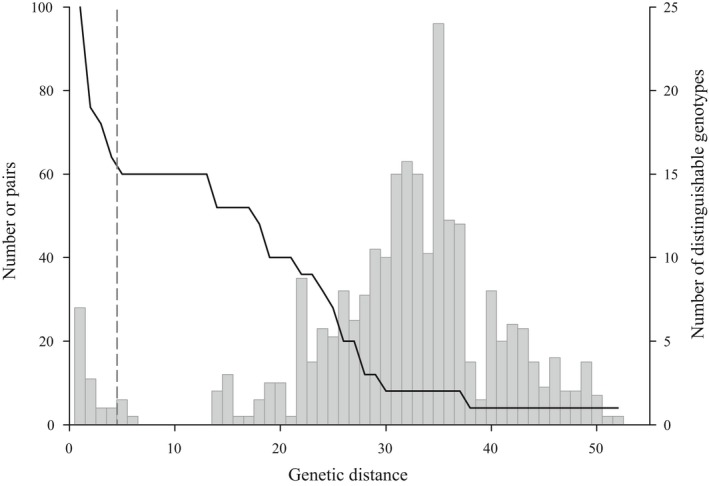
Frequency distribution of pairwise genetic distances (bars and left axis) based on ISSR markers between all *Opuntia microdasys* samples (*N* = 577) and number of distinguishable genotypes (solid line and right axis) under the selected threshold (dashed line). The threshold was selected according to the method of Meirmans and Van Tienderen (2004), and the pairwise distances and number of distinguishable genotypes to the left of the threshold correspond to a mismatch difference between clones of the same clonal linage. To the right of the threshold are the genotypes without errors and the clonal lineages chosen for the study

DNA amplification reactions were run in a total volume of 15 μl, with the following composition: 0.8 μM primer (for primers 817 and 842) or 0.6 μM (for primer 827), 1× PCR buffer (BIOGENICA), 2.0 mM MgCl_2_ (BIOGENICA), 0.2 mM dNTPs (Invitrogen), 1 U Taq polymerase (Amplificasa—BIOGENICA), and 2 μl (10–20 ng/μl) DNA of *O. microdasys* and purified water (Sigma). The amplification reactions were carried out in a PTC‐100 thermocycler (MJ Research) programmed with an initial denaturation of 4 min at 94°C; followed by 36 cycles of 30 s at 94°C, 45 s at 52°C, 2 min at 72°C, and 2 min at 72°C; and a final extension of 7 min at 72°C. Amplification products were separated on 1.4% agarose gels (0.5× Tris‐borate‐EDTA [TBE] buffer at 120 V for 4.5 h), stained with ethidium bromide (0.01%), and visualized and photographed under UV light. The molecular marker 1Kb Plus (Invitrogen) was used as a molecular weight standard. Digital images of the gels were obtained for each individual using LabWorks software 4.0 (UVP, Inc.). The banding pattern was subsequently transformed into a presence/absence matrix. To test the reproducibility of the band patterns, we re‐extracted and amplified DNA from a sample of 45 individuals taken from all parent plants from all clumps. If the banding pattern of the sampled replicates varied, we adjusted the conditions (i.e., purity and concentration of DNA) and, if necessary, diluted or repeated the extraction and amplification. Following the method of Bonin, Ehrich, and Manel (2007), we estimated error rate at the allelic level with the 45 repeated samples; the error rate for dominant markers was estimated from the number of phenotypic differences (band presence or absence) and the total number of comparisons (number of pairs = 96, threshold to assign multilocus genotypes of three bands, Figure 3, see below). The error rate estimated for this study was 3.15%. We also checked that no band exceeded a frequency of 1 − (3*/N*) according to the proposal of Lynch & Milligan (1994) for dominant markers.

#### 2.4.3 Genetic data analysis

When molecular markers are used to define multilocus genotypes (MLGs), individuals can be misallocated in two ways: (a) Genetically similar individuals are assigned as clones but in reality are from different MLGs; (b) dissimilar genetic individuals are assumed to be genets but are actually clones (Douhovnikoff & Dodd, 2003). Individuals could be misallocated into MLGs for three reasons: scoring errors in the banding patterns, PCR artifacts, and somatic mutations (Meirmans & Van Tienderen, 2004). These errors can create small differences between individual genotypes and thus bias individuals of the same clonal lineage (Douhovnikoff, McBride, & Dodd, 2005; Meirmans & Van Tienderen, 2004). To avoid this bias, we used the program GenoDive (Meirmans & Van Tienderen, 2004), which plots the differences between individuals against the frequency of individuals. A multimodal histogram frequently results, and a threshold must be chosen to determine the number of clonal lineages in the sample (Douhovnikoff & Dodd, 2003). For choosing a threshold for genetic differences among pairs of individuals and excluding scoring errors or small differences due to somatic mutations, Meirmans and Van Tienderen (2004) recommended using the valley between the first and the second peak as the threshold. In the present study, we thus set the threshold for determining differences at three bands (Figure 3).

We determined the probability that the detected genotypes are unique genets using *P*_dgen_ of Sydes and Peakall (1998), which is the probability of drawing a second copy of a particular genotype, given that one copy of this genotype has already been drawn from the population and assuming a population with sexual reproduction and random mating. *P*_dgen_ can be extended to the probability of drawing the same genotype *n* times as (*P*_dgen_)^*n*–1^. Further, *P*_dgen_ = ∏ *p*_*i*_, where *p*_*i*_ is the frequency of each locus in the multilocus genotype (Oddou‐Muratorio et al., 2011). On the basis of the genotyped lineages obtained using the selected threshold, banding patterns of ramets belonging to each clonal lineage were matched manually for further analysis.

For the total sample and for each clump of plants, we calculated the percentage of polymorphic bands (%P) and the number of private bands (PB) with the program GenALEX 6.4 (Peakall & Smouse, 2006, 2012). We also calculated the genotypic diversity index corrected for sample size:

$$R=\frac{G-1}{n-1},$$

where *G* is the number of genotypes identified in the sample and *n* is the sample size. *R* can have values between 0 and 1; *R* is 0 when all individuals are copies of the same genotype and 1 when all individuals have different genotypes (Dorken & Eckert, 2001).

An AMOVA for diploid binary data was used to determine the distribution of genetic variation among the levels of organization included in the study, clumps of plants and populations. This method calculates a matrix of Euclidean distances between pairs of individuals (Excoffier, Smouse, & Quattro, 1992). This test was performed with GenALEX 6.4 (Peakall & Smouse, 2006, 2012) after running 1,000 iterations.

We calculated *Φ*_PT_, which refers to a relation of the genetic variance among the populations relative to the total variance, but based on information of differences, matrix and differentiation among populations were calculated via AMOVA from haplotypes or dominant markers (Excoffier et al., 1992; Peakall & Smouse, 2006, 2012); and their interpretation is similar to *F*_ST_, values near 0 means no genetic structure and near to 1 high levels of differentiation between populations. To assess the most likely number of genetic clusters, we conducted two different Bayesian analyses of clustering, one with structure v. 2.3.4 (Pritchard, Stephens, & Donnelly, 2000a) and one with geneland v. 4.0.5. After an exploratory run, structure was run with the following parameters: admixture, allele frequencies correlated among populations, three populations with a location prior, 250,000 burn‐in and 500,000 MCMC iterations, *K* between 2 and 30, α inferred from data, and 20 repetitions for each value of *K*. The parameters admixture, allele frequencies correlated among populations, and location prior were used because they are recommended when local genotypes comprise alleles from many populations, which can obscure the identification of populations (Falush, Stephens, & Pritchard, 2003; Porras‐Hurtado et al., 2013; Pritchard, Stephens, & Donnelly, 2000a). Admixture was included in the model as populations of *O. microdasys* are relatively close and it is possible that actual genotypes represent a mixture of genetic composition of genotypes that come from different populations (Porras‐Hurtado et al., 2013), and we considered allele frequencies correlated as it helps to distinguish among genetic groups even if they are similar (Porras‐Hurtado et al., 2013). From the results, we selected the value of *K* using the structure harvester website (Earl & vonHoldt, 2012), which maximizes *ΔK*. Evanno, Regnaut, and Goudet (2005) defined *ΔK* as the second‐order rate of change in the likelihood function with respect to the number of genetic clusters (*K*). With geneland, we tested the values of *K* from 1 to 30, the number of iterations of MCMC was set to 500,000, with thinning of 100, coordinate uncertainty of 0.01, and a burn‐in period of 200 (Guillot, Estoup, Mortier, & Cosson, 2005). This criterion suggested *K* = 20, but 17 of the 20 putative clusters were empty *(*i.e., 17 ghost populations). Consequently, a second GENELAND analysis was performed with *K* fixed at three, 800,000 MCMC iterations, thinning of 1,000, and coordinate uncertainty of 0.01 and burn‐in of 100; 30 independent runs were done with the same parameters, and we selected the run in which no “ghost” populations occurred and obtained approximately the same estimates of individual population membership, *K*, and maps.

## 3 RESULTS

### 3.1 Evaluation of fruit dispersal

#### 3.1.1 Primary fruit dispersal

The mean directional angle (μ), mean resultant length of dispersal *(r)*, and the chi‐squared test for uniformity at which fruits fell in the primary dispersal experiment differed between populations (Table 2). The slope of each habitat affects the spatial pattern of fallen fruits. More fruits that dropped in the west quadrants fell with the highest frequencies to the SW, NW, and W at HPH (Appendix 1a), which resulted in a μ close to a west orientation (Table 2, Appendix 1). Three circular statistics support this finding: the highest values of mean resultant length of dispersal (*r* = 0.273) and the concentration (κ = 0.568) and the lowest standard deviation (*SD* = 92.32°). Quadrants NW > SW > NE > SE showed the highest frequency of fruits in BH (Appendix 1b). Nevertheless, the value of μ was close to NW (Table 2 and Appendix 1b; *r *=* *0.063, κ = 0.126, and *SD* = 134.76°). Finally, the frequency of fruits at IDH was more homogeneous between directions, with a slight increase in the frequencies to the NE, E, SE, and SW (Appendix 1c) and mean direction approximately to the E (Table 2 and Appendix 1c). The IDH population had the lowest frequency of fruits with a skewed orientation around a focal plant (*r *=* *0.03, κ = 0.061, and *SD* = 151.49°).

**Table 2:** Circular statistics of the experiment to simulate primary dispersal of fruits of *Opuntia microdasys* in three populations (BH: Bajada, HPH: Hill‐piedmont, IDH: Interdune) from the southern Chihuahuan Desert

Model	Population		
Variable	HPH	BH	IDH
Mean direction (μ)	259.441°	313.032°	76.23°
Mean resultant length of dispersal (*r*)	0.273	0.063	0.03
Concentration (κ)	0.568	0.126	0.061
Circular standard deviation (*SD*)	92.329°	134.762°	151.494°
Chi‐squared test (uniform, χ²)	822.4	339.81	23.46
Chi‐squared test (*p*)	<1E‐12	<1E‐12	0.001
Weighted statistics			
Weighted mean vector (WMV)	262.496	324.264	122.791
Length of WMV (in m)	31.721	5.158	2.104
Length of WMV (*r*, scaled 0–1)	0.041	0.032	0.011

The correlation between direction and fruits dispersal distance was analyzed with circular–linear correlations and was significant for all populations (HPH, *r *=* *0.393, *p *<* *0.0001; BH, *r *=* *0.207, *p *<* *0.0001; IDH, *r *=* *0.107, *p *<* *0.0001). The mean dispersal distance of fruits differed between populations (ANOVA, *F*_2,27_ =* *9.53, *p *<* *0.001) and quadrants (*F*_7,9556_ =* *69.31, *p *<* *0.001), and the interaction between factors was significant (*F*_14,9556_ =* *67.31, *p *<* *0.001). The interaction reflects the influence of specific quadrants in each habitat; for example, quadrants with west orientations determined the fate of fruits in HPH (Appendix 2). The longest mean dispersal distance reached by fruits occurred in HPH (58.4 cm), followed by 32.18 cm in IDH, and the minimum distance was found in BH (29.9 cm; Appendix 2).

#### 3.1.2 Marking and tracking fruits

**Figure 4:**
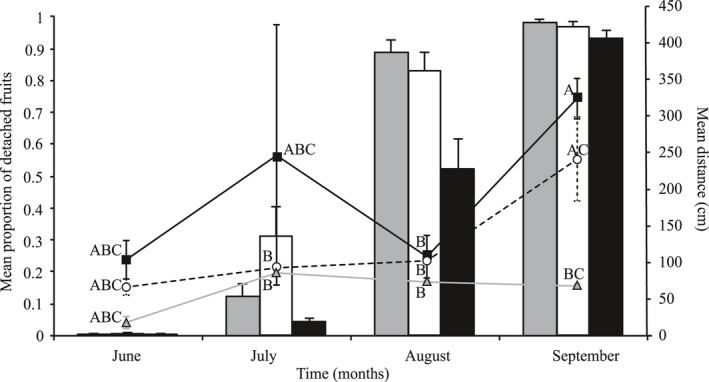
Mean proportion (Bars +SE) of detached fruits and mean distance (Lines ±*SE*, secondary *y*‐axes) traveled by fruits of *Opuntia microdasys* over the four sampling periods. Bars represent proportion of detached fruits (+*SE*) in each habitat Bajada = gray bars, Hill‐Piedmont = white bars, and Interdune = black bars. Lines represent the distance traveled by fruits in each habitat: 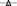 = Bajada, 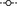 = Hill‐Piedmont, and 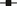 = Interdune. Same letters indicate groups that did not differ significantly (Tukey's post hoc test)

The monthly proportion of fruits detached from the parent plant was statistically significant (full model: goodness of fit Pearson value χ^2^ = 6150.8, *df* = 108, *p *<* *0.001, and deviance χ^2^ = 6577.3, *df* = 108, *p *<* *0.001, AIC = 152.9). The proportion of detached fruits did not differ between populations (χ^2^ = 1.92, *df* = 2, *p* = 0.382), but the proportion did differ among months (χ^2^ = 453.5, *df* = 3, *p *<* *0.001), and an interaction existed between months and population (χ^2^ = 19.08, *df* = 6, *p* = 0.004). The proportion of detached fruits increased during the fruiting season, from 0.003 to 0.004 at the first count in June to 0.82 ≅ 1 at the last count (Figure 4, Bars). The population with the highest proportion of detached fruits was BH, with 88% since the third count. In contrast, the proportion in IDH was less than HPH in the third count and higher for the last count (Figure 4, Bars).

Fruit dispersal distance differed between populations (*F*_2,189_ = 3.617, *p *<* *0.0287). In the IDH, fruits disperse the farthest (average 182 cm), followed by HPH (106 cm), then BH (75 cm). Dispersal distance also differed among months (*F*_3,7061_ = 12.627, *p *<* *0.0001), with the longest distance in the fourth count (273 cm) and the shortest in the first count (63 cm; Figure 4, lines, secondary *y*‐axes). The interaction between factors was also significant (*F*_6,6433_ = 4.925, *p *<* *0.0001). Fruits in the IDH moved farthest, and fruits in BH always moved the shortest distance (Figure 4, lines).

The full model for the proportion of fallen fruits at each stage of maturity was significant (full model goodness of fit Pearson value: χ^2^ = 12,764.4, *df* = 456, *p *<* *0.0001, deviance value: χ^2^ = 10,053.4, *df* = 456, *p *<* *0.0001, AIC = 454.07); the factors population (χ^2^ = 4.1, *df* = 3, *p* = 0.2491) and stage of fruit maturity (χ^2^ = 2.0, *df* = 2, *p* = 0.3673) were not statistically significant, but month had a significant effect (χ^2^ = 194.4, *df* = 3, *p *= <0.0001) as fruits mature (Appendix 3a). The interaction of population with the maturity stage of fallen fruits was statistically significant (χ^2^ = 22.01, *df* = 6, *p *<* *0.0001), because most fruits fell in the first or second count at BH and IDH and abortion rate was constant over time at HPH. Also, the interaction of the month and the stage of maturity of fruits differed (χ^2^ = 65.31, *df* = 9, *p *<* *0.0001) because the highest proportion of fallen ripe fruits peaked in the second or the third count, unripe fruits peaked in the first count and that proportion decreased toward mid‐season (Appendix 3a). One other difference is that in IDH, numerous fruits were not found in the first and the last count (Appendix 3a).

We found significant differences in the proportion of fruits with different fates (full model goodness of fit Pearson value: χ^2^ = 19,244.07, *df* = 570, *p *<* *0.0001, deviance value: χ^2^ = 15,687.65, *df* = 570, *p *<* *0.0001, AIC = 572.68). The proportion of fruits did not differ between populations (χ^2^ = 0.975, *df* = 2, *p* = 0.6141), but there were differences among months (χ^2^ = 56.78, *df* = 3, *p *<* *0.001) and fates (χ^2^ = 28.383, *df* = 4, *p *<* *0.001), with a significant interaction between population and fate (χ^2^ = 49.88, *df* = 8, *p *<* *0.001) and between months and fate (χ^2^ = 66.321, *df* = 12, *p *<* *0.001). The fates with the highest proportions of fruits were the parent and lost fruit fates (Appendix 3b) in all populations. The number of fruits found in the parent fate was highest in the first count of the fruiting period and decreased with time. The number of lost fruits was highest in the fourth count, although in IDH the maximum lost had occurred by the second count (Appendix 3b). The exposed fate was consistent in all three populations but variable over time (Appendix 3b). Finally, the *Neotoma* nest and other fates had the lowest proportions. The *Neotoma* nest fate was particularly common in the IDH, especially in the first count (Appendix 3b).

### 3.2 Genetic structure of clumps of plants and populations

Of the 577 individual ramets that were genotyped with the three selected ISSR primers (Table 1), we identified 115 different clonal lineages (genotypes) with GenoDive using a three‐band threshold of differences between genotypes (Figure 3).

In most clumps of plants, we found clonal individuals. The population with the highest genotypic diversity was HPH (*R* = 0.435), followed by IDH (*R* = 0.222) and BH (*R* = 0.087; Table 1). Plant clump IDH8 had the highest genotypic diversity (*R* = 1) and the fewest members (only three ramets). In HPH, the highest values of *R* corresponded to two clumps of plants (HPH3 and HPH6; *R* = 0.917 and 0.719, respectively; Table 1). On the other hand, in BH and IDH, most clumps of plants had lower genotypic diversity (0 in several cases), indicating a predominantly clonal composition (Table 1). The values of *P*_dgen_ were very low for all populations (BH = 0, HPH = 1.6e^−38^, IDH = 5.13e^−89^), which suggests that the assignment of clones is robust.

In agreement with HPH having the highest genotypic diversity, HPH also had the highest percentage of polymorphic bands and private bands (%P = 82.44%, PB = 23), followed by IDH (%P = 65.95%, PB = 22) and BH (%P = 57.71%, PB = 7, Table 1). At the level of clumps of plants, there was no clear pattern; the values of %P and PB varied between clumps of plants, and none of the clumps had high values for either polymorphic loci or private alleles (Table 1). The AMOVA between habitats—without considering clumps—showed the highest percentage of variation within populations (Table 3) and only 21% of the variation between habitats. This result means that the differentiation of populations was moderate to high (*Φ*_PT_ = 0.21). The AMOVA that included the clumps and populations also highlighted the fact that almost all the variation was found between clumps (81%, *Φ*_PT_ = 0.92, Table 3), with very little variation between populations (11% of variation) or within clumps (8%).

**Table 3:** AMOVA analysis (a) excluding the factor clump to compare between populations and (b) including both clumps and populations. % = Percentage of the total genetic variation found in each clump and population

Source	*df*	Variance	%
(a) Population level			
Among populations	2	5.815	21
Within populations	574	21.941	79
Total	576	27.757	100
(b) All levels			
Among populations	2	2.999	11
Among clumps	27	22.482	81
Within clumps	547	2.136	8
Total	576	27.616	100

**Figure 5:**
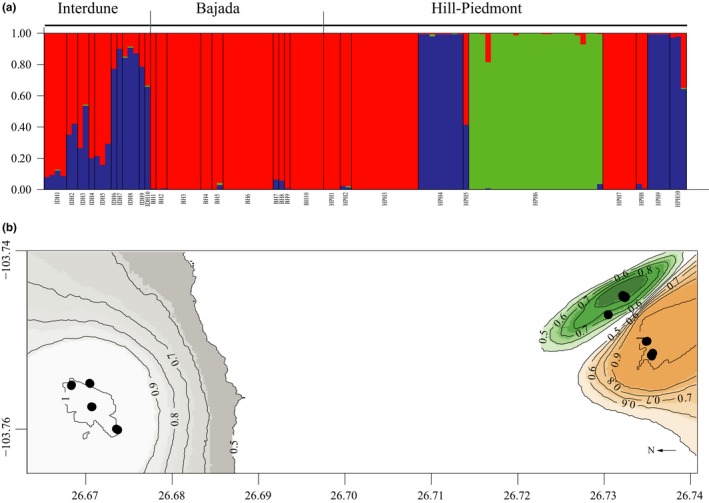
Assignment of genotypes of three populations of *Opuntia microdasys* (Bajada, Hill‐Piedmont, and Interdune) to genetic clusters based on Bayesian methods. (a) structure assignment to *K* = 3. (b) geneland assignment to *K* = 3, with different colors for each cluster and with density probabilities of membership to each cluster indicated by lines

We tested two Bayesian methods of assignment of individuals into genetic clusters: structure (Pritchard, Stephens, & Donelly, 2000b) and geneland (Guillot et al., 2005). The same number of clusters (*K* = 3) was chosen in both analysis, but assignment of individuals differed between the two methods. The three clusters predicted by structure were represented by individuals from all three populations in different proportions. In BH, the proportion of individuals in cluster one (red cluster in Figure 5a) was 0.99; in the HPH, three genetic clusters were present in similar proportions (red cluster = 0.4, green cluster = 0.37, and blue cluster = 0.23, Figure 5a); and in the IDH genetic clusters, one and three were predominant (red cluster = 0.55 and blue cluster = 0.44, Figure 5a). In the geneland analysis, the genetic clusters corresponded closely to the spatial distribution of individuals. All individuals from IDH were assigned to one cluster (white cluster in Figure 5b). All individuals but one (BH1) from BH were assigned to another cluster (tan cluster in Figure 5b), and all individuals from HPH, with the BH1, were assigned to a third cluster (green cluster in Figure 5b).

## 4 DISCUSSION

Field experiments and molecular genetics were both useful for estimating dispersal of clonal and sexual diaspores for *O. microdasys* and to determine the consequences of dispersal on genotypic diversity for clumps of plants and between populations.

On one hand, because plantlets are easily traceable dispersal units, direct observation of dispersal was plausible for this species and gave us an accurate estimate of short dispersal distances, but the estimates were not accurate for long distances (Bullock et al., 2006; Nathan & Muller‐Landau, 2000; Nathan et al., 2003). However, we found other abiotic factors that affected dispersal. For example, microtopography modified the trajectory and distance reached by fruits, at the population with the steepest slope, fruits reached longer distances and produce clumps of plants with intermingled genotypes (HPH). In contrast, in the population with the shallowest slope (IDH), the fruits accumulated near the source and produced monoclonal clumps. Movement of clonal propagules is influenced by gravity and slope (Chambers & MacMahon, 1994). Few fruits moved longer distances (i.e., 100 m), following the same leptokurtic curve proposed for seed dispersal (Nathan & Muller‐Landau, 2000; Willson, 1993). Furthermore, during primary dispersal, fruits move on average only 1 m, even under the influence of a slope, a common phenomenon in species dispersed by gravity (Chambers & MacMahon, 1994; Nanami et al., 1999; Pairon et al., 2006). The influence of orientation, slope angle, and gravity on the formation of clone or seed clumps has been previously quantified (Pairon et al., 2006). For example, *Podocarpus nagi* seeds forms clumps under the canopies of large female trees (Nanami et al., 1999), and 95% of the seeds from *Prunus serotina* fall within 0–5 m of the source (Pairon et al., 2006).

The importance of abiotic factors such as river water flow and the hydrologic regime determines the site of deposition of postrelease propagules of *Betula fontinalis* (Merritt & Wohl, 2002). Short‐distance dispersal deposits the propagules where the offspring will establish; Palleiro (2001) commonly found offspring within a 90‐cm radius under the crown of *O. microdasys* individuals. We expected that the morphological traits of the fruits should confer higher mobility, but this type of propagule shares some traits with other clonal propagules: They are larger than seeds, have shorter dormancy periods and lack a specialized dispersal mechanism, which usually leads to a clumped distribution (Eckert, 2002). Nonetheless, secondary agents increased the mean dispersal distance reached by fruits. We found evidence of postdispersal of fruits by mammals (painted fruits within the nests of *Neotoma albigula*). The association of fruits with packrat nests and the greater mobility of fruits as the duration of exposure to secondary vectors increased are evidence of the redistribution of fruits and secondary dispersal. Water streams formed after heavy rain is another vector of secondary dispersal that modifies the distance reached by fruits, water moved fruits 440 m (M. Mandujano, unpublished data). Propagules of species that undergo secondary dispersal are commonly redistributed by a second agent (Bohning‐Gaese, Gaese, & Rabemanantsoa, 1999; Griffith & Forseth, 2002), which generally increases the dispersal distance (Nathan & Muller‐Landau, 2000). Helsen, Verdyck, Tye, and Van Dongen (2009) suggested that finches carry *Opuntia echios* seeds long distances between different islands in the Galapagos Islands. Another example of bird dispersal of *Opuntia* is found in the Karoo, South Africa (Dean & Milton, 2000); crows (*Corvus capensis*) move seeds of *Opuntia ficus‐indica* and may be the most important vector for the range expansion of this *Opuntia* species.

On the other hand, indirect methods that use molecular markers are well developed and are used to evaluate effective dispersals (i.e., dispersal plus establishment events; Cain et al., 2000, Levin, et al., 2003), determine the number and distance of migrants per generation, and the degree of genetic structure and differentiation of populations (Cain et al., 2000; Carrillo‐Angeles et al., 2011; Levin et al., 2003; Manel et al., 2005; Pritchard, Stephens, & Donelly, 2000a). Indirect methods, however, are focused on effective dispersal (Cain et al., 2000) and exclude all the reproductive structures that dispersed but have not established or survived.

Unlike species with linked clonal growth, in which the ramets are spatially clumped (Charpentier, 2002), *O. microdasys* produces unlinked ramets from unripe fruits (clonal propagules) with traits that we had expected to provide greater mobility and, consequently, longer dispersal distances. This kind of pseudo‐viviparity is a common phenomenon found in other species of Cactaceae such as *Opuntia* spp. and *Cylindropuntia* spp. (Bravo‐Hollis, 1978; Fuentes Pérez, 2008; Negron‐Ortiz & Strittmatter, 2004; Nobel, 2002; Palleiro et al., 2006; Piña et al., 2007; Vázquez‐Delfín et al., 2005). Therefore, we expected new plantlets to establish away from the parent plant, resulting in intermingled genotypes. Nevertheless, the response was not straightforward, genotypic diversity in some clumps of plants of BH was very low or even completely clonal, while other clumps were genetically diverse (HPH, Table 1). This pattern reflects the high clonal recruitment that occurs in the clumps of this population and corresponds with the amount of clonality reported by Palleiro et al. (2006). The low genotypic diversity found in IDH could reflect the importance of each phase of dispersal in the genetic configuration of the clumps of plants; actually, the short distance reached by fruits during primary dispersal would favor the formation of clumps of ramets from the same genet, and the fruits that dispersed longer distances or to unsuitable habitats (e.g., *Neotoma* nests) apparently failed to establish new offspring. The population with the highest genotypic diversity was HPH, for which slope was the main factor influencing primary dispersal, and HPH was the population with the highest percentage of sexual recruitment (Palleiro et al., 2006).

Most genetic variation occurred within a population or among clumps (AMOVA, Table 3); the highest genetic differences were found between clumps, and the value of *Φ*_PT_ indicated high to moderate genetic differentiation between the studied populations. This pattern is dissimilar to other clonal species with low values of differentiation among populations as in *Potamogeton pectinatus* in which Abbasi, Afsharzadeh, and Saeidi (2017) found values of *Φ*_PT_ = 0.11 and the highest genetic variation located within populations (89%). In a study with *Bromus ircutensis*, a clonal grass, found *F*_*ST*_ values ranged from 0.118 to 0.15% and 87% of the genetic variation within the populations. structure analysis assigned some individuals from all three populations to one genetic cluster (Figure 5a); consequently, we could not recognize a characteristic genotypic pattern for each population, and we found that most of the genotypic variability occurred within populations and between clumps, supporting the idea that although the establishment of new ramets occurs mostly in the area under a parent plant, there is migration and genet flow between populations (Porras‐Hurtado et al., 2013). Most genotypes were overrepresented, and migration rates of clonal propagules were low, based on a sample of 10 clumps of plants from each population, so this scale allowed us to detect the migration of clonal propagules. Nonetheless, migration rates might in fact be greater than determined here if the sampling strategy had been designed to collect samples of most of the genotypes present in the populations. The assignment of genotypes to genetic clusters in structure suggests that migration could play a more significant role than we detected with field experiments; in fact, assignation analysis reveals the actual scope of dispersal abilities of propagules and the effective dispersal (Figures 2 and 5). structure identified a predominant cluster for all three populations (Figure 5a, cluster in red), but a blue cluster that was present only in IDH and HPH. In contrast, by adding the spatial location of clumps, geneland separated the populations, and just one clump of BH was assigned with all clumps of HPH. The difference between these two analyses could be due to the effect of adding spatial data, as all other factors of organization of this study (i.e., offspring under focal plants, clumps, clumps in quadrants, plots of populations, and populations, Figure 2) were the same.

High migration rates or gene flow either by unripe fruits or seeds could be the reason for the spatial genetic structure; that is, most genetic variation occurred within population and among clumps. In addition, gene flow is also limited by pollen dispersal in *Opuntia microdasys*, based on the behavior of the primary pollinator (Piña et al., 2007), in addition to either clonal or sexual offspring dispersal. Furthermore, other examples of similar migration rates have been shown for very well‐structured populations (Gélin et al., 2017; Yu, Han, Tian, & Liu, 2011) or for small, isolated populations (Kim & Chung, 1995). An extreme example was found in *Opuntia echios* on the Galapagos Islands, in which no clonal individuals were found in a sample of 444 individuals collected in 22 localities (Helsen, Verdyck, & Van Dongen, 2011). The scale at which levels of variation were observed and the offspring recruitment reported (Palleiro et al., 2006) are evidence indicating a pattern of repeated seedling recruitment (Eriksson, 1992) at the population level, but with clonal recruitment playing an important role at a more local scale (clumps and genet survival).

Although *O. microdasys* was able to recruit using the three possible pathways in all the studied populations, the percentage of recruitment of each type of offspring differed in each population (Palleiro et al., 2006). This pattern is a clear indicator not only of the capacity of the species to produce clonal propagules and sexual diaspores in a variety of habitats, but also of the ecological conditions that limit the sites where each type of dispersal unit was more successful at establishment and the needs of dispersal units to arrive at these safe sites. The clonal propagules and sexual diaspores must be dispersed to reach these sites (Hroudova & Krahulcova, 1996; Nathan & Muller‐Landau, 2000), and the nature of that dispersal determines the pattern of recruitment and distribution of the genetic and genotypic variation in the neighborhood and population. The reproductive strategy of *O. microdasys*, maintaining clonal genotypes that can exploit favorable sites in limiting habitats for long periods, often results in monoclonal patches (Gélin et al., 2017; van Groenendael, Klimes, Klimesova, Hendriks, & Van Groenendael, 1996), as occurred in the BH and IDH habitats. However, this strategy incurs a cost by increasing the levels of geitonogamy and limiting the pollen flow between different genotypes (Charpentier, 2002; Zhang & Zhang, 2007). Such pollen limitation has been studied in *Maianthemum bifolium*, a clonal self‐incompatible species, for which fruit set is affected in populations with a low level of genotypic diversity (Honnay, Jacquemyn, Roldán‐Ruiz, & Hermy, 2006). In a self‐incompatible species such as *O. microdasys* (Piña et al., 2007), in which the offspring frequently establish under the parent plant (Palleiro et al., 2006), the aggregation of ramets necessarily leads to some level of geitonogamy (Charpentier, 2002; Zhang & Zhang, 2007), mainly when there is a spatial autocorrelation of genets over short distances (<20 m; Nathan et al., 2003). It is expected that geitonogamy acts as a positive feedback mechanism that favors sexual failure, abortion, and clonality, which thus increase geitonogamy.

The combination of ecological field experiments with molecular genetics experiments allowed us to assess dispersal in a complex system with species with different structures of dispersal and with many levels of organization. Our results suggest that the genetic structure of clumps of plants in part is due to the limited mobility of clonal propagules, bounded by primary dispersal and environmental restrictions for their establishment. This dispersal process leads to an unequal distribution of dispersal unit types at each population (with limited dispersal of clonal propagules and more long‐distance dispersal of sexual diaspores), generating monoclonal and intermingled clumps, but with a level of migration over longer distances that allowed some differentiation among populations.

## CONFLICT OF INTEREST

None declared.

## AUTHOR CONTRIBUTIONS

All authors contributed with planning, designing, analyzing, and writing of the manuscript. The study is part of the PhD project of GME and is included within a long‐term project of population biology of *Opuntia* spp. of MCM. Other details: EGM; Israel Carrillo‐Angeles (ICA); MCM; and Jordan Golubov did the field work. EGM, ICA, and MCM performed the molecular experiments. MCM and Daniel Piñero edited the MS as advisors and provided financial and laboratory facilities to carry out molecular experiments.

## DATA ACCESSIBILITY

Data of sampling locations, ID of each plant and population, ISSR genotype of each individual, and UTM locations of each sampled genotype will be available in the Dryad Digital Repository.

## Figures and Tables

**Figure A1:**
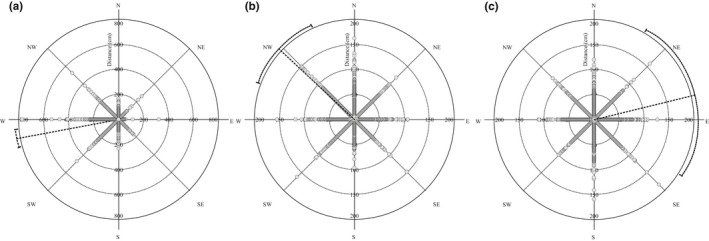
Scatterplot of fruits of *Opuntia microdasys* that fell on cardinal and intercardinal quadrants and distances traveled by fruits around a focal plant at each population: (a) Hill‐Piedmont, (b) Bajada, and (c) Interdune. Dotted line is the mean direction angle (μ) and circular standard deviation (*SD*)

**Figure A2:**
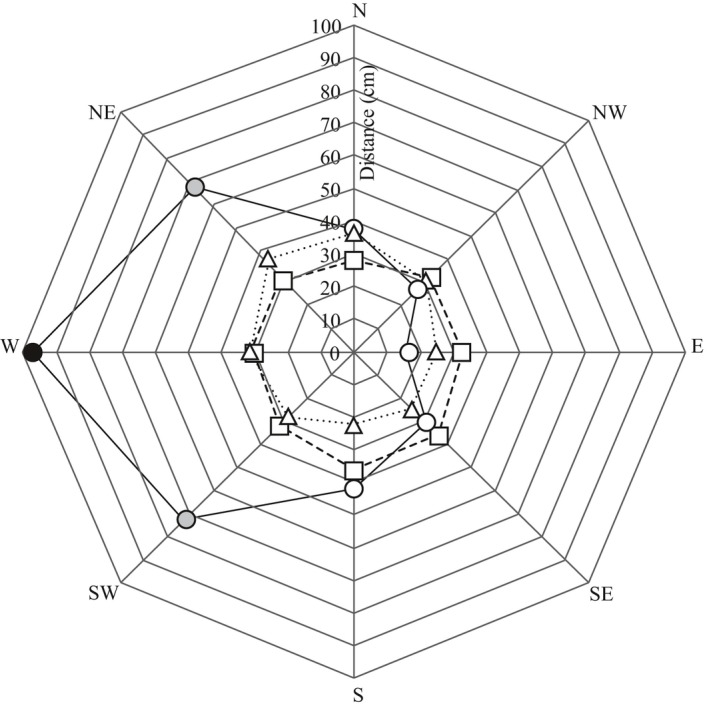
*Opuntia microdasys* mean fruit dispersal distance (cm) at eight cardinal and intercardinal quadrants. Symbol shading indicates when more (black), fewer (gray), or the same (white) number of fruits fell into quadrants than expected by chance. Population key: 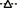 = Bajada, BH, 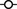 = Hill‐Piedmont, HPH, and 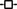 = Interdune, IDH

**Figure A3:**
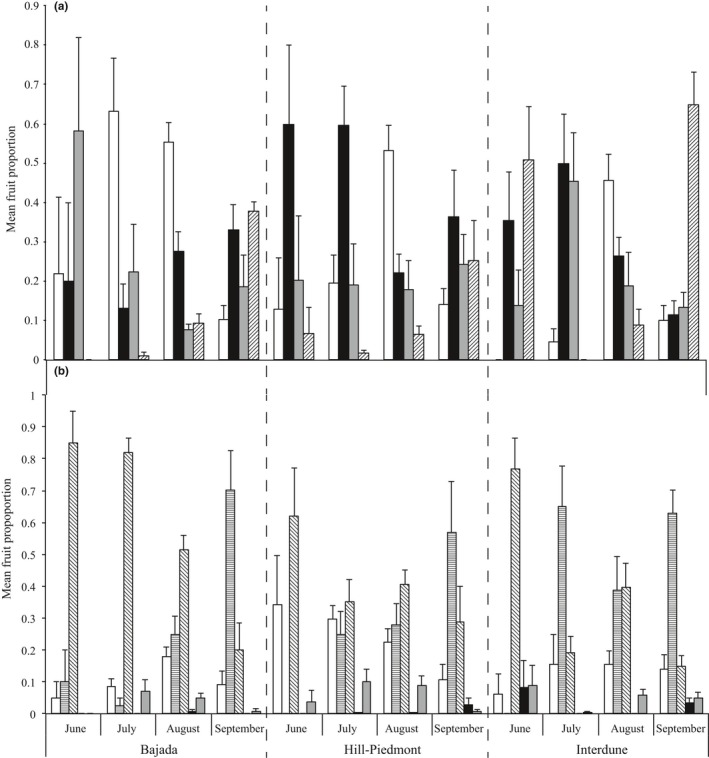
Mean proportion (+*SE*) of fallen fruits of *Opuntia microdasys* over time grouped by population: (a) type of fruit found (white = ripe, black = unripe, gray = aborted, hatched = partial fruit). (b) Final fates of fallen fruits over time grouped by population during the track/mark experiment of fruit dispersal (white bars = exposed in bare areas; horizontal hatched bars = lost; diagonal hatched bars = under crown of mother plant; black bars = *Neotoma albigula* nest; gray bars = other plants)
